# Nanosilica Extracted from Hexafluorosilicic Acid of Waste Fertilizer as Reinforcement Material for Natural Rubber: Preparation and Mechanical Characteristics

**DOI:** 10.3390/ma12172707

**Published:** 2019-08-23

**Authors:** Van-Huy Nguyen, Cuong Manh Vu, Hyoung Jin Choi, Bui Xuan Kien

**Affiliations:** 1 Department for Management of Science and Technology Development, Ton Duc Thang University, Ho Chi Minh City 700000, Vietnam; 2 Faculty of Applied Sciences, Ton Duc Thang University, Ho Chi Minh City 700000, Vietnam; 3 Center for Advanced Chemistry, Institute of Research and Development, Duy Tan University, Da Nang 550000, Vietnam; 4 Department of Polymer Science and Engineering, Inha University, Incheon 22212, Korea; 5 Faculty of Natural Sciences, Electric power University, 235 Hoang Quoc Viet St., Bac Tu Liem Dist, Hanoi 100000, Vietnam

**Keywords:** natural rubber, nanosilica, mechanical property, fertilizer plant, hexafluorosilicic acid, waste water

## Abstract

Nanosilica particles are extracted from waste water containing a hexafluorosilicic acid discharged from Vietnamese fertilizer plants as an effective way not only to reduce waste water pollution but also to enhance the value of their waste water. Amorphous nanosilica particles are produced with diameters ranging from 40 to 60 nm and then adopted as a reinforcing additive for natural rubber (NR) composites. Morphological, mechanical, rheological, and thermal behaviors of the nanosilica-added NR composites are examined. Especially, mechanical behaviors of nanosilica-filled NR composites reach the optimum with 3 phr of nanosilica, at which its tensile strength, hardness, and decomposition temperature are improved by 20.6%, 7.1%, and 2.5%, respectively, compared with the pristine vulcanized NR. The improved mechanical properties can be explained by the tensile fractured surface morphology, which shows that the silica-filled NR is rougher than the pristine natural rubber sample.

## 1. Introduction

While nanosilica is becoming one of the most widely used nanomaterials for many industries, with an annual growth of 5.6%, silica is used as an important filler for rubber in a range of products, such as tires and other industrial materials, because it increases its mechanical durability, heat resistance, shrinkage, thermal expansion, and stress [1,2,3,4]. In addition, it improves the wear resistance of rubber based composites by replacing a soft matrix with a hard inorganic filler. Therefore, silica-based polymer nanocomposites have many exciting features for their many applications in the automotive, electronics, marine, and other industries. Many researchers have used silica as a reinforcement material for rubber-based composites [5,6,7,8,9].

Mechanical characteristics of the rubber can be enhanced using nanosilica only or combined with other fillers [10,11]. In particular, high-quality silica also has an absolute advantage in the manufacture of household products (fashion footwear soles, rubber mattresses, and others) or medical rubber (gloves, boots). In the pharmaceutical industry, silica is used as a carrier for some proprietary medicines [12,13,14]. In the organic chemical industry, silica acts as a catalyst for some organic reactions, helps acceleration rates, and improves reaction yields [15,16,17,18]. To meet the increasing demand for silica in industrial applications, many studies focused on the fabrication of silica from different sources [19,20,21,22,23,24,25,26,27,28]. In addition to the many common sources, such as silane compounds of Na_2_SiO_3_, hexafluorosilicic acid (H_2_SiF_6_) becomes a potential economical candidate for the production of silica. Hexafluorosilicic acid is a by-product from the fertilizer industry, produced in huge quantities annually [29]. On the other hand, it is toxic and harmful to the environment, requiring either chemical treatment or conversion to a highly economical product [30]. For example, in Vietnam, Lam Thao Fertilizers and Chemicals, the largest production company of fertilizers with an estimated capacity of approximately 850,000 tons/year, produces approximately 30,000 tons of hexafluorosilicic acid annually. The production of hexafluorosilicic acid in the fertilizer production line can be explained as follows: First, fluorapatite (Ca_5_(PO_4_)_3_F (calcium fluorophosphate)) is reacted with either H_2_SO_4_ or HNO_3_ to generate HF according to the following reactions:

Ca_5_(PO_4_)_3_F + 5H_2_SO_4_ + nH_2_O --> 3H_3_PO_4_ + 5CaSO_4_·nH_2_O + HF

Or

Ca_5_(PO_4_)_3_F + 10HNO_3_ --> 3H_3_PO_4_ + 5Ca(NO_3_)_2_ + HF

Obtained HF reacts with SiO_2_, which exists in the composition of the raw materials, to form SiF_4_ gas:

4HF + SiO_2_ --> SiF_4_ + 2H_2_O

The collection of H_2_SiF_6_ is usually performed by an absorption method of gaseous SiF_4_ in a water scrubber.

3SiF_4_ + 2H_2_O = 2H_2_SiF_6_ + SiO_2_

Many groups have reported its utilization, such as in silica recovery [30,31,32,33,34,35,36,37]. Dragicevic and Hraste [38] prepared silica from the neutralization of fluosilicic acid with ammonia while Sarawade et al. [25] used Na_2_CO_3_ to recover mesoporous silica with a large surface area from waste H_2_SiF_6_ from the fertilizer company. Hexafluorosilicic acid was further adopted as a silica source to fabricate SZM-5 as a trans-alkylation catalyst [29]. Cicala et al. [39] synthesized amorphous silicon alloys from fluorinated gases by plasma deposition, while Guzeev et al. [40] produced zircon and zirconium tetrafluoride with silicon tetrafluoride and zirconium dioxide as a raw material. Liu et al. [41] also synthesized titanium containing Mobil Composition of Matter No. 41 (MCM-41) with the industrial H_2_SiF_6_ and applied for cyclohexene epoxidation reaction.

We report a simple method of recovering amorphous silica nanoparticles from hexafluorosilicic acid waste and their application as a reinforcing filler in natural rubber (NR) with enhanced mechanical and thermal characteristics in this study. Both fabricated nanosilica and nanosilica-added NR are characterized. These efforts could not only reduce the environmental pollution of hexafluorosilicic acid wastes, but also enhance the value of waste from a fertilizer plant as an inorganic filler of NR. Note that of the total cost for the final product in factory including the cost for raw material, equipment, energies, and waste water treatment etc., the cost for waste water treatment grows higher as a result of the government policy and type of the waste water. This results in the higher price of final products, reducing their competitiveness. In case of Vietnamese fertilizer plants, the by-product of H_2_SiF_6_ with its emission rate is about 35,000 tons per year. With its highly toxic and corrosive characteristics, the H_2_SiF_6_ solution could threaten the environment by contaminating rivers and oceans.

Therefore, we strongly believe that the production and utilization of nanosilica in this study could become a reliable and sustainable solution for dealing with waste water from fertilizer plants environmentally as well as economically.

## 2. Experimental Procedures

### 2.1. Materials

The hexafluorosilicic acid was collected from Lam Thao Fertilizers and Chemicals JSC (Phu Tho, Vietnam) and used as a raw resource for the production of nanosilica. The concentration of the hexafluorosilicic acid solution was 13 wt.% along with a very low concentration of Fe_2_O_3_ (0.01–0.02 wt.%) and P_2_O_5_ (0.001–0.01 wt.%). A 25 wt.% ammonia solution was purchased from Xilong Scientific Co. (Shantou, China). The NR (NR-Standard Vietnamese Rubber-3L) was purchased from Phuoc Hoa Rubber Co. (Binh Duong, Vietnam). We adopted 2, 2, 4-trimetyl-1, 2-dihydroquinolin (RD), N-cyclohexyl-2-benzothiazole sulfenamide (CBS), and 2, 2’-dibenzothizole disulfide (DM), (RongCheng K&S Chem., Rongcheng, China) as vulcanizing accelerators [42]. Stearic acid, zinc oxide, and sulfur were purchased from Sigma-Aldrich (St. Louis, MO, USA).

#### 2.1.1. Recovery of Nanosilica

**Figure 1:**
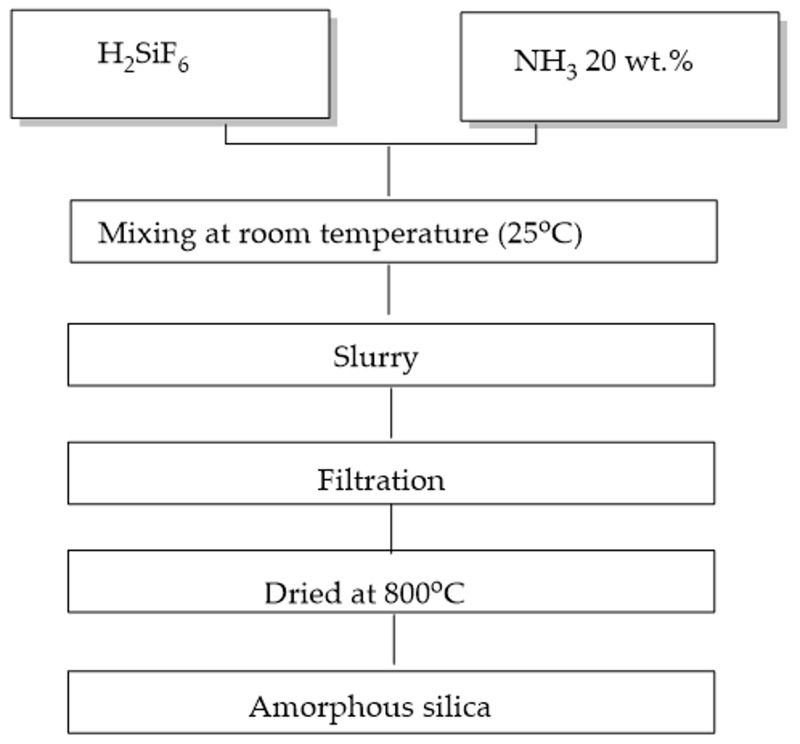
The processing of fabrication of nanosilica from hexafluorosilicic acid.

In the first step, pristine solutions of both hexafluorosilicic acid and ammonia were diluted with distilled water to 10 wt.% and 20 wt.%, respectively. In the second step, 200 g of 10 wt.% hexafluorosilicic acid and 110 g of 20 wt.% ammonia solution were mixed in a glass reactor vessel with a mechanical stirrer at 200 rpm for 12 h at 25 °C to obtain a slurry of nanosilica. Subsequently, the silica nanoparticles were collected with a vacuum filter under atmosphere 200 mg Hg and cleaned a couple of times with distilled water until they reached a neutral pH value. The resulting products were dried using a vacuum oven at 800 °C for 3 h and cooled to 25 °C before obtaining the final product. Figure 1 provides details of this processing.

The material characteristics of the obtained nanosilica, such as chemical structure, crystallinity, and particle size were scrutinized by Fourier transform-infrared spectroscopy (FT-IR), X-ray diffraction (XRD), and transmission electron microscopy (TEM), respectively.

#### 2.1.2. Rubber Compound Fabrication

The NR compounds both with and without nanosilica were fabricated following the formulations given in Table 1. Initially, the NR, ZnO, stearic acid, paraffin, and nanosilica were put into an internal mixer (Brabender Plasti-corder 350s, Brabender GmbH & Co. KG, Berlin, Germany) at different compositions depending on the sample number and mixed well at 50 °C with a rotor speed of 50 rpm for 1 h [42]. Further mixing was accomplished with a two-roll mill once three vulcanizing accelerator chemicals and sulfur were added. The rubber compounds were either used for examining curing characteristics or vulcanized to determine their mechanical properties.

### 2.2. Measurements

The moving die rheometer (Monsanto, MDR2000P, St. Louis, MO, USA) was used to determine the curing characteristics of NR compounds. About 5 g of rubber compound was inserted into the geometry of two parallel rotating disks at 150 °C at a frequency of 100 rpm. After processing was completed, the cure curve with many characteristics such as max. torque (M_H_), min. torque (M_L_), scorch time (t_2_), and 90% cure time (t_90_) were acquired.

In order to prepare the samples for tensile testing, a sheet of about 2 mm thickness was vulcanized in a molding test press (Gotech, GT7014H, Taichung, Taiwan) at 150 °C and a 40 kgf/cm^2^ pressure for a respective cure time, t_90_, which was estimated from the MDR 2000P measurement [42].

The tensile test was performed using an Instron universal testing machine according to ASTM D412-93 at room temperature (~25 °C). Tensile strengths and elongations at break were estimated from stress-strain curves and averaged values from five-time rerun measurements for each sample were obtained [34]. The Shore A hardness of the samples was evaluated following the ISO 7619-1:2010. Thermogravimetric analysis (TGA) was performed with a TGA Q50 (TA Instruments, New Castle, DE, USA) according to the ASTM D3850-94 method. Approximately 10–20 mg of vulcanized samples were loaded onto an open platinum pan, and then heated from 25 to 600 °C under a nitrogen environment at a fixed heating rate of 10 °C/min. The fracture surfaces of the fabricated nanocomposites were examined with a scanning electron microscope (SEM) (SEM JEOL 5510, JEOL, Tokyo, Japan) at 10 kV accelerating voltage.

The Mooney-Rilvin equation was used to determine the crosslinking density of the vulcanates based on the following stress-strain behavior [42]:

$$\sigma =2(\lambda -\frac{1}{\lambda^{2}})(C_{1}+\frac{C_{2}}{\lambda })$$

where *σ*, *λ*, *C*_1_, and *C*_2_ are the tensile stress, the strain, and constants, respectively. The *C*_1_ and *C*_2_ constants were determined from the slope and intercept of the curve of *σ*/(*λ* − *λ*^−2^) versus 1/λ. Finally, the crosslinking density was obtained from the following equation:

2*C*_1_ = ρkT

where ρ is the cross-linking density, k is the Boltzmann constant, and T is the absolute temperature.

## 3. Results and Discussion

**Figure 2:**
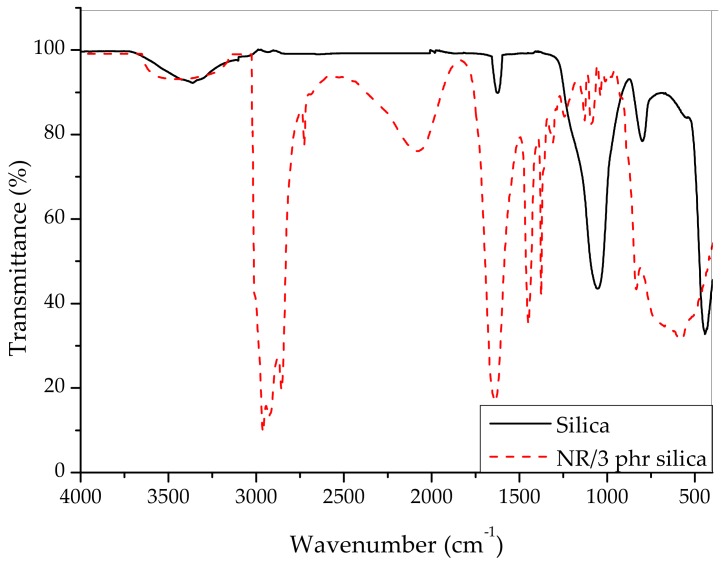
FT-IR spectra of fabricated nanosilica and natural rubber (NR)/3 phr silica composite.

Initially, the chemical structure of fabricated silica nanoparticles was examined with the help of an FT-IR spectroscopy (Perkin Elmer, Waltham, MA, USA) as shown in Figure 2. The silica nanoparticles exhibited a distinctive absorption peak at 1000–1100 cm^−1^, which was ascribed to a stretching vibration of Si–O–Si bonding. Another distinctive absorption peak also appeared at 1630 cm^−1^, which was allocated to a bending vibration by way of H–O–H in the water molecules. An additional absorption peak was further detected at 3400 cm^−1^, which was due to a stretching vibration of the hydroxyl group, confirming the existence of hydroxyl groups in the silica surface. Figure 2 also showed the FT-IR spectrum of vulcanized natural rubber filled with 3 phr silica composite. It is seen that the asymmetrical stretching vibration of Si–O–Si and the hydroxyl of silica in the NR/SiO_2_ composite appeared at 1088 cm^−1^ and 3403 cm^−1^, respectively.

**Figure 3:**
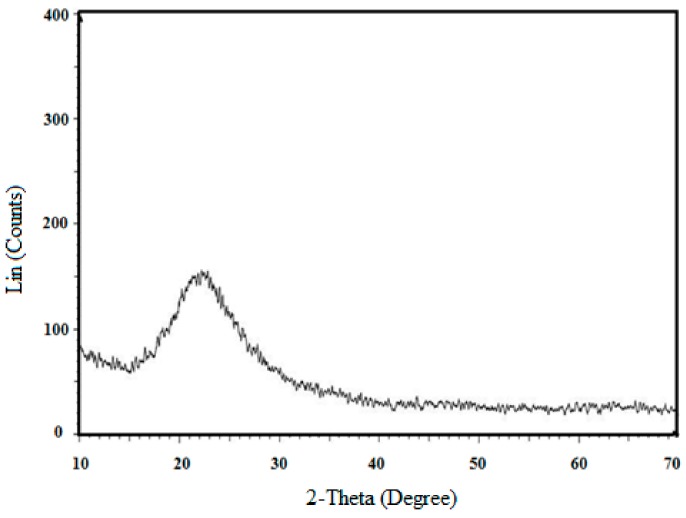
XRD pattern of nanosilica.

**Figure 4:**
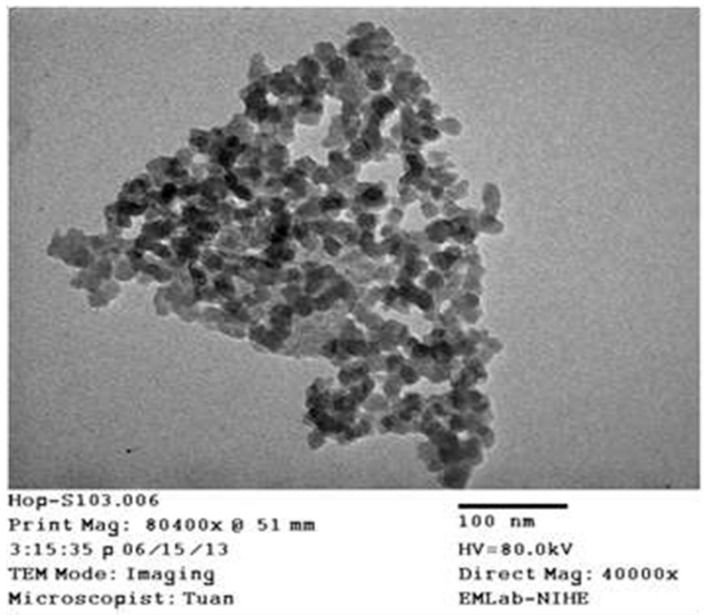
TEM of nanosilica.

Regarding the crystalline structure of the samples, the powder XRD pattern in Figure 3 indicated a broad peak at 22° of 2θ, revealing the amorphous nature of the nanosilica particles. Furthermore, the TEM image of nanosilica in Figure 4 showed its diameter of 40–60 nm.

**Figure 5:**
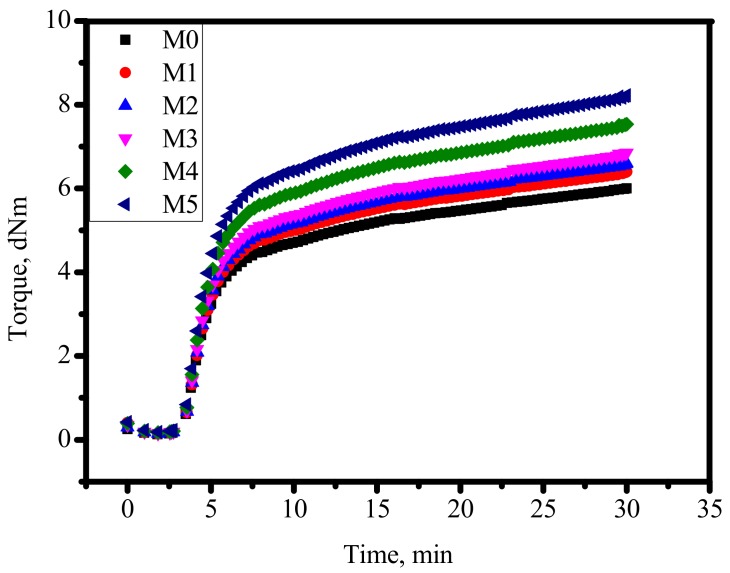
Cure curves of the investigated rubber compounds taken at 150 °C (black line—M0; red line—M1; blue line—M2; pink line—M3; olive line—M4; green line—M5).

Table 2 lists the main curing characteristics of the fabricated NR compounds that were obtained from the cure curves (Figure 5), in which both t_2_ and t_90_ increased with increasing silica nanoparticle content in the NR. In general, the minimum torque (M_L_) in Figure 5 is associated with a shear viscosity of the blend, while the maximum torque (M_H_) has a relation to the elastic stiffness of vulcanized samples. The results implied that with addition of nanosilica, both M_L_ and M_H_ values increased, meaning that the viscosity or stiffness of vulcanized NR increased with the presence of nanosilica particles. The difference between maximum torque and minimum torque, (M_H_-M_L_), which was obtained from the dynamic shear modulus test, corresponds indirectly to the crosslinking density of the vulcanization. The results indicated that the (M_H_-M_L_) exhibited the same tendency to the maximum torque and increased with increasing nanosilica loading as a result of increased cross-linking of the NR with the presence of nanosilica particles.

**Figure 6:**
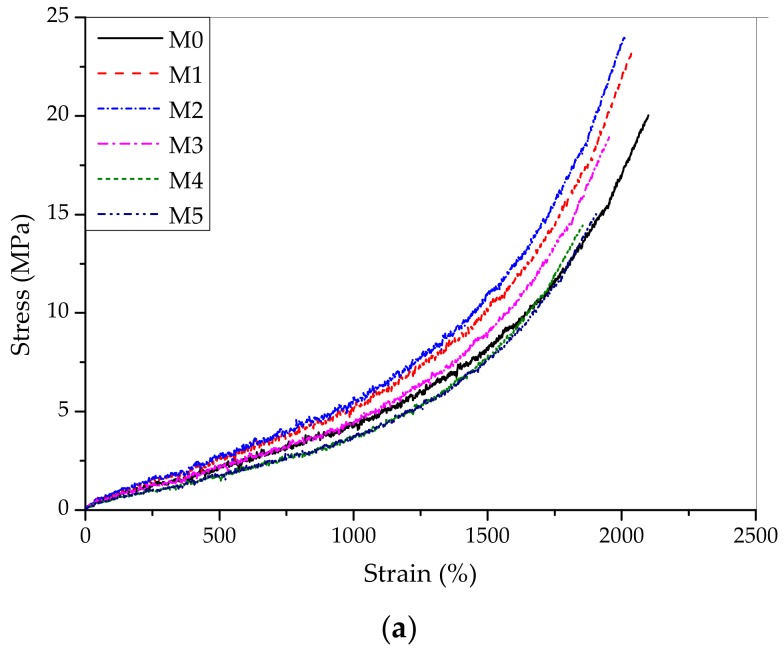

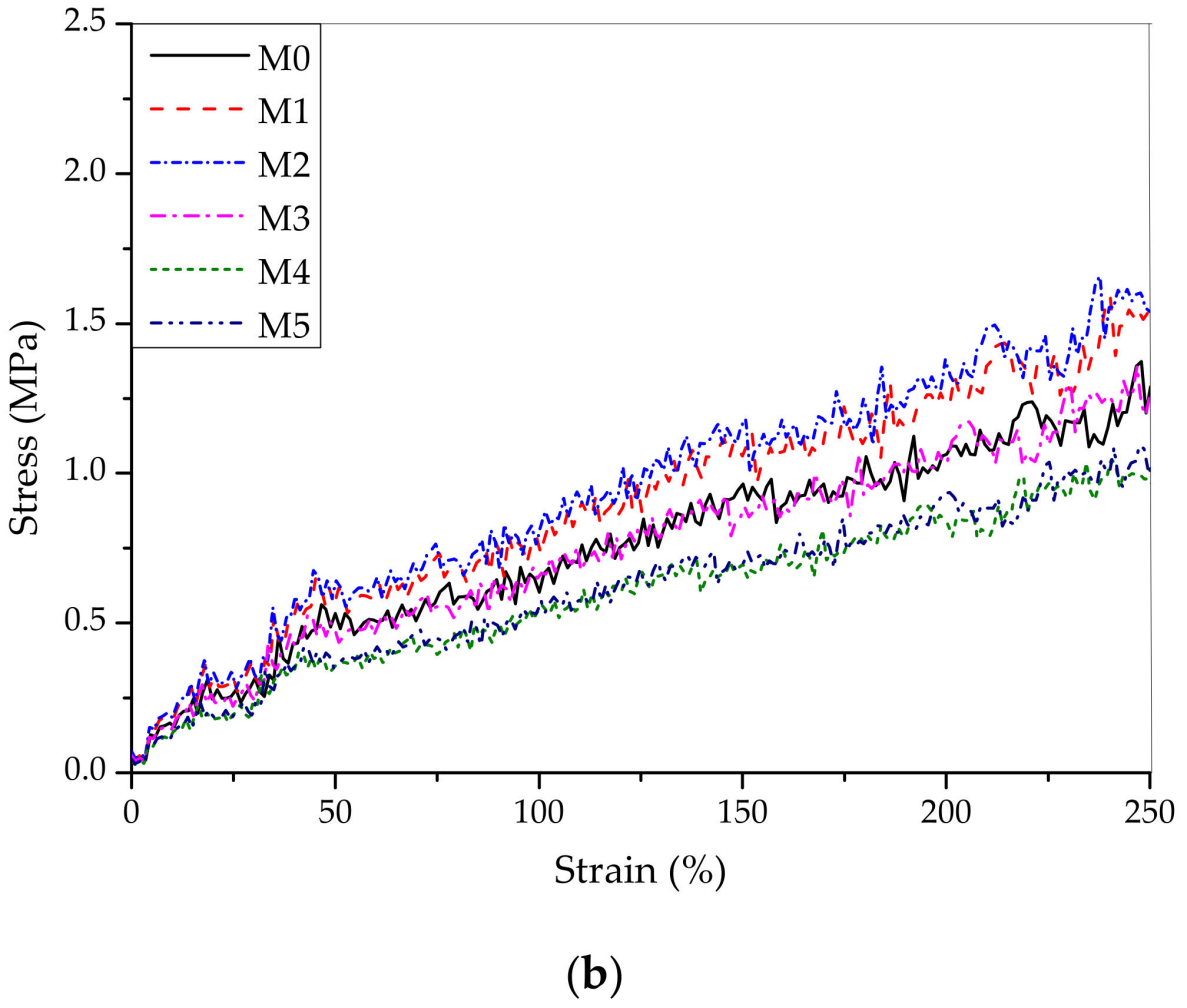
(**a**) Typical stress-strain of cured samples (Black line—M0; red line–M1; blue line—M2; pink line—M3; olive line—M4; orange line—M5). (**b**) The bottom figure is the one expanded in the initial stage;

Figure 6 presents typical stress-strain curves of the nanosilica-reinforced NR composite materials. All samples exhibited the deformation-forced crystallization characteristics with the rapidly raised sharp slopes when the strain reached more than 1500% [36,43,44]. Meanwhile, the tensile stress and slope curves increased with increased contents of silica and reached the optimal properties at 3 phr of silica filled NR. The details of the mechanical characteristics are provided in Table 3.

The tensile strength exhibited the decreasing trend when the silica content exceeded 3 phr. Although the elongation at break decreased with increased silica concentration, the hardness of the NR showed the same trend with the tensile strength. Both changes in the tensile strength and elongation at break for different nanosilica loadings have a relationship with a cross-linking density of the cured NR composites. That of the NR increased with silica concentration, resulting in an increase of the tensile strength and a decrease of the elongation at break due to the decreased slippage among the molecular chains. The H-bond between silica particles and rubber chain prevented the mobility and slippage of the rubber chain. On the other hand, the fact that tensile strength only increased with silica contents up to 3 phr may be due to the agglomeration of nanosilica at a higher content. The crosslinking densities of samples with name M0, M1, M2, M3, M4, M5 were 7.82 × 10^5^, 8.06 × 10^5^, 8.19 × 10^5^, 8.22 × 10^5^, 8.36 × 10^5^ and 8.45 × 10^5^ mol/cm^3^, respectively. The trend of crosslinking density is the same as with the trend of M_H_ and hardness. The hardness also increased with increased silica content. The incremental increase of crosslinking density resulted in the decreasing of elongation at break because of the prevention of the slip between each NR molecule. In addition, more energy was needed to break the linkage between each rubber molecule as the result of increased tensile strength.

**Figure 7:**
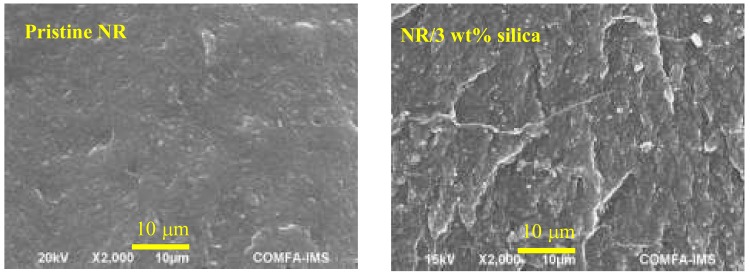
SEM images of the fracture surface of pristine natural rubber and 3 phr silica filled natural rubber.

The SEM image results of the tensile fractured surface of the NR composites both in the absence and presence of 3 phr of nanosilica were presented in Figure 7. These results indicated that the fractured surface of the 3 phr silica-filled NR was observed to be rougher in comparison with the pristine sample. Therefore, extra energy was required compared to the case of the smooth fractured surface of the pristine NR. This result is in agreement with the tensile results shown in Figure 6.

**Figure 8:**
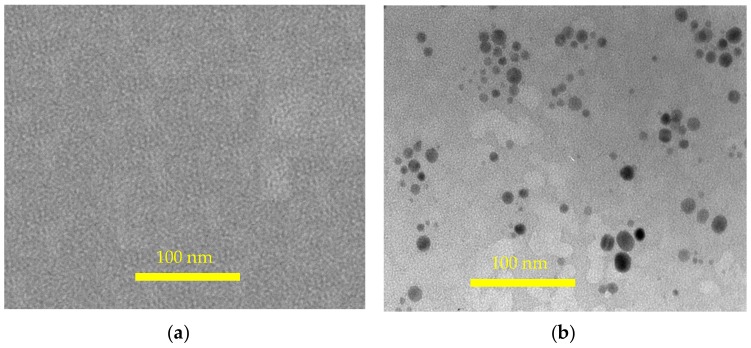
TEM images of pristine natural rubber (**a**) and 3 phr silica filled natural rubber (**b**).

The TEM images of both the natural rubber and 3 phr silica filled natural rubber are shown in Figure 8. The pristine NR sample had no silica particles, while the silica particles existed in the modified NR with nano scale from 20–60 nm.

**Figure 9:**
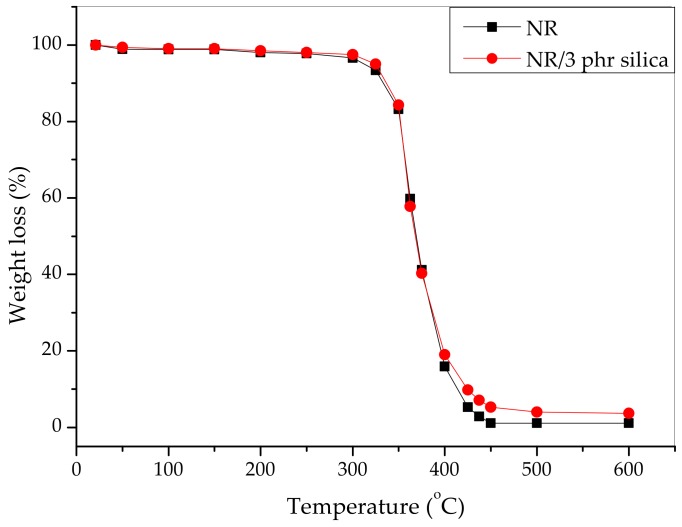
TGA of cured natural rubber (NR) (black dot line) and 3 phr of nanosilica filled natural rubber (red dot line).

The thermal degradation of both the pristine NR and the 3 phr nanosilica filled NR composites was examined in terms of the weight loss (%) as a function of temperature, as shown in Figure 9.

Thermal degradation of the NR can be explained via various processes such as chain-scission of the polymers, and breakage of the cross-linked portion. The silica filled NR composite exhibited a 2.5% higher decomposition temperature in comparison with the pristine NR because of the existence of H-bonds between silica particles and rubber chains with higher thermal stability. The thermal degradation of both pristine and silica filled NR occurs through the degradation of isoprene units at around 368 °C. The presence of nanosilica induced the higher residues of nanocomposite when compared with pristine NR due to the presence of inorganic filler, which is more thermally stable than NR. The TGA results demonstrate that the nanosilica filled NR showed only a very slightly higher thermal stability than that for the NR. The initial temperature decomposition, the maximum decomposition temperature, and residue of silica modified NR and pure NR were 268.2 °C, 368.2 °C, 1.1% and 266.8 °C, 366.3 °C, 3.7%, respectively. The entire thermal degradation of the nanocomposite can be explained by the two-step process. First of all, the rubber chains and cross-linking were deteriorated into smaller parts. In the second step, the smaller parts in the first step continuously degraded into volatile products and disappeared. The residual char was higher for the nanosilica-filled sample.

**Figure 10:**
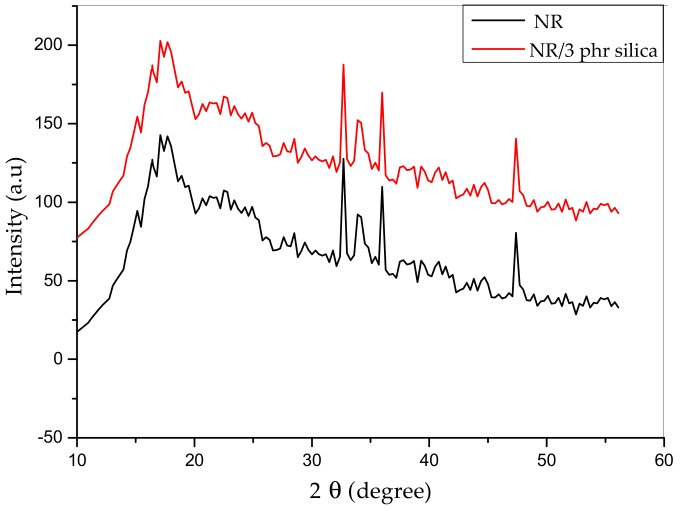
XRD spectra of vulcanized NR and NR/3 phr silica.

Figure 10 shows the XRD patterns of vulcanized pure NR and NR/3 phr silica nanocomposite. The broad diffraction peak around 20° is the noncrystalline structure of NR, while the diffraction peaks between 30°–50° are assigned to ZnO particles in the vulcanizates. None of the samples show obvious characteristic peaks of graphite or silica, indicating that silica particles are homogeneously dispersed in the rubber matrix. These results indicated that the silica did not keep the amorphous structure when embedded in natural rubber.

## 4. Conclusions

In this study, amorphous silica nanoparticles (between the sizes of 40–60 nm) were extracted from hexafluorosilicic acid waste produced by the Vietnamese fertilizer industry via a precipitation process. The production and utilization of the nanosilica by-product could become a reliable and sustainable solution for dealing with waste water from fertilizer plants environmentally as well as economically, regarding the waste water treatment. The resulting silica nanoparticles were then adopted as the filler for NR. The tensile strength of 3 phr silica-added NR nanocomposites increased by 20.6% compared to that of pristine NR. The elongation at break decreased with increased filler content and the hardness of the filled sample increased with increasing nanosilica content. The filled sample also exhibited better thermal resistance than the pristine sample due to the presence of nanosilica.

## Figures and Tables

**Table 1:** The composition of silica/natural rubber compounds.

Ingredients (phr)	M0	M1	M2	M3	M4	M5
Natural Rubber Zinc Oxide Stearic Acid Parafin RD CBS DM Sulfur Silica	100.0 5.0 3.0 1.0 2.5 1.5 0.5 2.0 0.0	100.0 5.0 3.0 1.0 2.5 1.5 0.5 2.0 1.0	100.0 5.0 3.0 1.0 2.5 1.5 0.5 2.0 3.0	100.0 5.0 3.0 1.0 2.5 1.5 0.5 2.0 5.0	100.0 5.0 3.0 1.0 2.5 1.5 0.5 2.0 7.0	100.0 5.0 3.0 1.0 2.5 1.5 0.5 2.0 10.0

**Table 2:** Curing properties of the silica/rubber compounds.

Samples	Curing Properties				
	Minimum Torque, M_L_ (dN·m)	Maximum Torque, M_H_ (dN·m)	△M = M_H_ − M_L_ (dN·m)	Scorch Time, t_s2_ (min:s)	Cure Time, t_90_ (min:s)
M0	0.135	6.000	5.865	4:11	15:89
M1	0.144	6.400	6.256	4:25	16:15
M2	0.148	6.600	6.452	4:39	16:41
M3	0.154	6.850	6.696	4:41	16:83
M4	0.169	7.535	7.366	4:54	16:92
M5	0.185	8.220	8.035	4:60	17:01

**Table 3:** Mechanical properties of cured NR/silica compounds.

Samples	Mechanical Properties		
	Tensile Strength (MPa)	Elongation at Break (%)	Hardness (Shore A)
M0	20.02	2100.12	38.51
M1	23.18	2036.85	39.72
M2	24.15	2014.52	41.24
M3	18.93	1953.77	42.51
M4	15.13	1909.49	43.22
M5	14.45	1855.26	45.06
